# Prevalence and factors associated with cancellation and deferment of elective surgical cases at a rural private tertiary hospital in Western Uganda: a cross-sectional study

**DOI:** 10.11604/pamj.2021.39.139.24667

**Published:** 2021-06-17

**Authors:** Bienfait Mumbere Vahwere, Franck Katembo Sikakulya, Robinson Ssebuufu, Soria Jorge, Xaviour Francis Okedi, Shaban Abdullah, Patrick Kyamanywa

**Affiliations:** 1 Department of Surgery, Kampala International University, Kampala, Uganda,; 2 Faculty of Medicine, Catholic University of Graben, Butembo, Democratic Republic of Congo

**Keywords:** Elective surgery, cancellation, deferment, rural setting, Uganda

## Abstract

**Introduction:**

the cancellation of elective surgery is still a worldwide challenge and this is associated with emotional and economical trauma for the patients and their families as well as a decrease in the efficiency of the operating theatre. This study aimed at determining the prevalence and factors associated with cancellation and deferment of elective surgery in a rural private tertiary teaching hospital in Western Uganda.

**Methods:**

a cross-sectional study design was conducted. Data was collected from 1^st^ July 2019 to 31^st^ December 2019. Patients scheduled for elective surgery and either cancelled or deferred on the actual day of surgery were included in the study. Statistical analysis was done using STATA version 15.

**Results:**

four hundred patients were scheduled for elective surgery during the study period, among which 90 (22.5%) were cancelled and 310 (78.5%) had their surgeries as scheduled. The highest cancellation of elective surgical operations was observed in general surgery department with 81% elective cases cancelled or deferred, followed by orthopedic department 10% and gynecology department 9%. The most common reasons for cancellation were patient-related (39%) and health worker-related (35%) factors. Other factors included administrative (17%) and anesthesia related factors (9%). Cancellation was mainly due to lack of finances which accounted for 23.3% of the patients, inadequate patient preparation (16.6%) and unavailability of surgeons (15.5%). Major elective surgeries were cancelled 1.7 times more than minor electives surgeries [adjusted prevalence ratio 1.7 (95%CI: 1.07-2.73) and p-value: 0.024].

**Conclusion:**

cancellation and deferment of elective surgeries is still of a major concern in this private rural tertiary hospital with most of the reasons easily preventable through proper scheduling of patients, improved communication between surgical teams and with patients; and effective utilization of available resources and man power.

## Introduction

The cancellation of a scheduled list for elective surgery is still a worldwide challenge [1]. Cancellation of surgery refers to failure to realize a scheduled surgical procedure [2]. Cancellation can be avoidable and non-avoidable and the latter is seen as the commonest in developing countries with a range of 10-45% and 0.37-28% in developed countries [3]. Abrupt cancellation of elective surgery leads to emotional trauma and financial loss for such patients and their families [4].

The reasons for cancellation of elective surgery are not exhaustive and vary from one hospital to another [5]. Alterations in patients´ medical status and emergency cases are the most common unavoidable causes of cancellation [2,5]. However, inadequate preoperative evaluation, scheduling errors, unavailability of human resources and lack of equipment are the avoidable reasons for late cancellation of patients from the operation list [5].

The elective case cancellation rate on the day of surgery is an indicator of operating theatre efficiency [6]. Although there is no consensus on the acceptable cut off for case cancellation rates when defining efficient operating theatres, less than 5% is generally recommended [5,6]. In Uganda, studies conducted in the public health sector found that the prevalence of cancellation of elective surgical procedures was between 23.9% to 28.8%, with orthopedic surgery having the highest cancellation rate [7,8]. Two-thirds of the factors causing cancellations were facility-related and more than 50% of all cancellations were potentially avoidable [8] and yet in rural area especially in private hospitals, data is still limited on cancellation and deferment of elective surgery. This study focused on cancellation of elective surgeries in a rural tertiary hospital in Western Uganda with the aim of determining the prevalence and contributory factors to cancellation in order to generate strategies to reduce the rate of cancellation of elective surgeries.

## Methods

**Study design:** this was a cross sectional study carried out from 1^st^ July 2019 to 31^st^ December 2019 at a rural private tertiary teaching hospital in Western Uganda.

**Setting:** the study was done in operating theatres of Kampala International University Teaching Hospital (KIU-TH), a private nonprofit teaching hospital located in Bushenyi district in Western Uganda. KIU-TH has a capacity of 400 beds with 5 operating rooms where major and minor surgical procedures are performed. The Department of General Surgery has general surgeons, residents and intern doctors. Three days per week are set aside for elective surgeries (Monday, Wednesday and Friday) while the other days are reserved for booking of patients in the outpatient clinics. Each theatre day has at least 2 surgeons and residents assigned for elective operations. The orthopedic ward has specialist orthopedic surgeons and orthopedic clinical officers and each orthopedic surgeon has a specific day per week for elective bone cases.

The Department of Obstetrics and Gynecology has specialist obstetricians and gynecologists, residents and intern doctors; each gynecologist and a group of residents have a specific day per week for elective surgery and similar organization exists for the Department of Ear, Nose and Throat (ENT) and the Dental Department. The Department of Ear, Nose and Throat (ENT) has otorhinolaryngologists who are assigned for operation of elective cases on each theatre day and lastly the Dental Department has 2 dental surgeons assigned for elective dental cases on each theatre day. KIU-TH has anesthesiologists and anesthetic officers who also perform the pre-anesthetic assessments of all patients on the definitive theatre list a day prior to surgery.

**Participants:** all patients scheduled for elective operation constituted our study population. All patients on the theatre list submitted to the theatre team a day prior to the date of operation and were cancelled on the actual day of the operation were included in our study.

**Data sources/measurement:** the reason of cancellation of the surgery as given by the surgeon and recorded on the hard copy of the theatre list was captured using a structured questionnaire. The reasons for cancellation were grouped into: anesthesia related, surgeon related, administrative and patient related.

### Variables

**Socio-demographic factors:** age, sex, address, highest educational level attained and profession.

**Types of surgeries:** minor surgery was defined as any invasive operative procedure in which only skin or mucus membranes and connective tissue was incised or resected, with minimal bleeding of less than 500 ml and patient did not require admission after the operation [9]; Major surgery: was defined as any invasive operative procedure in which a more extensive resection was performed, for example a laparotomy with organs removed, or with normal anatomy altered during which bleeding of more than 500ml was expected and the patient was admitted for at least a day after operation [9].

**Health workers related reasons:** transfer to another service: patient needed to be managed by a surgeon of a specific specialty; decision that the patient is not fit for surgery, for example: i) patient didn´t need an operation but chemotherapy/radiotherapy; ii) no need for secondary closure because the wound is healing by secondary intention; iii) patient needed manual reduction and immobilization with a plaster of Paris (POP); iv) bowel not well-prepared, dirty wound and wrong diagnosis. Surgeon not available; needed further investigation: for example computerizing tomography scan (CT scan), prostatic specific antigen (PSA), liver function tests (LFTs), renal function tests (RFTs), barium enema, cysto-urography (CUG).

**Anesthesia related reasons:** decision made by the anesthetic team to cancel the surgical procedure. For example, that the patient has high blood pressure, low hemoglobin, patient with acute respiratory infection, patient not adequately starved, inaccessibility of the peripheral vein for administration of drugs and any other condition which can compromise the life of a patient if given anesthesia.

**Patient related reasons:** financial constraints; patient declined surgery; patient didn´t turn up.

**Administrative related reasons:** lack of equipment such as feeding tubes, Humby´s knife, breathing system for children; no space for operation: theatre contaminated after a dirty emergency case like gas gangrene, interruption by an emergency operation; lack of oxygen, shortage of blood in the blood bank, lack of sundries or any other drug.

**Data processing and analysis plan:** data was exported from Excel into STATA version 15 for statistical analysis. Categorical variables were presented using frequencies, percentages and continuous variables were presented using means, standard deviations (SD) and inter-quantile range (IQR). The categorical variables were analyzed using Chi-square and/or t-test for categorical variables and continuous variables respectively, to study associations with cancellation of surgery. Poisson regression was used for cancelled surgery as the dependent variable and all other variables taken as the independent variables. We did bivariate and multiple regressions and presented crude and adjusted prevalence-rate ratios (a.PR) to estimate the strength of the associations. The prevalence-rate ratios were used on the assumption that the prevalence of cancellation of surgery was common as it was over greater than 15%. All the variables with a p-value of <0.2 in the bivariate analysis were selected for inclusion into the multivariable model analysis.

**Ethical approval and consent to participate:** this study received approval from the management of Kampala International University Teaching Hospital in Western Uganda. Permission to access records was obtained from the hospital administration of KIU-TH and data was collected anonymously. There was no interaction with patients and patient consent was deemed not necessary. Only statistical data was extracted from the hospital records.

## Results

A total number of 94 theatre lists of elective operations from the Department of General Surgery, ENT, Dental, Orthopedic and Gynecology were submitted to the theatre team a day before the scheduled operation in the period under review. The average number of patients per theatre list was 5 (range 1-10) and the total number of patients who were scheduled for surgery which was 400. The Department of ENT and Dental did not cancel any elective surgery during this study.

**Figure 1:**
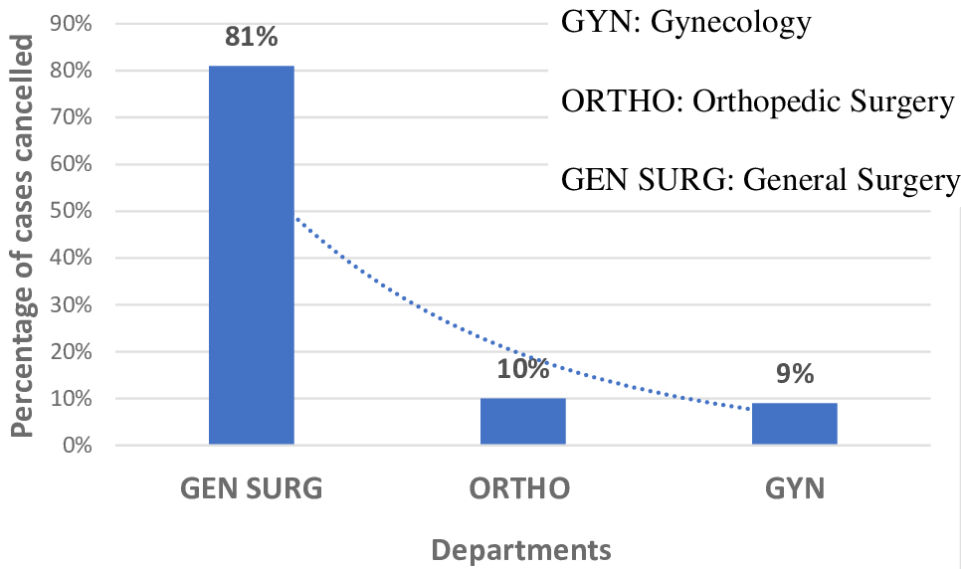
cases cancelled among elective surgery in each department at Kampala International University Teaching Hospital, June-December, 2019

Among the 400 patients who were scheduled for surgical operations; the majority 310 (77.5%) were operated while 90 patients (22.5%) were cancelled or deferred. Of the cancelled elective surgeries, pre-anesthetic review was performed on 46.6% of the patients while 54.4% did not get pre-operative assessment. During our study most of the patients who were cancelled were from surgery department 81% compared to orthopedic (10%) and obstetrics and gynecology (9%) departments (Figure 1).

**Socio-demographic characteristics of the study population:** from the 400 patients planned for surgery, 103 (25.8%) were aged 20 years and below, 237 (59.2%) between 40 and 60 years and 60 (15.0%). The age mean was 36 years. The majority of patients were male 241 (60.2%). Most of the patients were from rural area 247 (61.8%); most patients were self-employed (peasant farmer) 189 (47.3), followed by unemployed 72 (18.0%), student 72 (18.0%) (Table 1).

**Table 1:** socio-demographic characteristics of the study population

Variable	Total (percent)	Surgery cancelled	
		**Yes**	**No**
Sample size, n (%)	400 (100.0)		
Age in years, mean (SD)	36.7 (22.2)		
Age in years, median (IQR)	32.0 (34.0)		
**Age group in years, n (%)**			
20 and below	103 (25.8)	16 (15.5)	87 (84.5)
21 to 40	35 (25.5)	102 (74.5)	21 to 40
41 to 60	237 (59.2)	58 (24.5)	179 (75.5)
61 and above	60 (15.0)	16 (26.7)	44 (73.3)
**Sex, n (%)**			
Male	241 (60.2)	52 (57.8)	189 (61.0)
Female	159 (39.8)	38 (42.2)	121 (39.0)
**Residence, n (%)**			
Rural	247 (61.8)	51 (56.7)	196 (63.2)
Urban	153 (38.2)	39 (43.3)	114 (36.8)
**Profession, n (%)**			
Private	45 (11.3)	9 (10.0)	36 (11.6)
Government	22 (5.5)	4 (4.4)	18 (5.8)
Self	189 (47.3)	45 (50.0)	144 (46.5)
Unemployed	72 (18.0)	17 (18.9)	55 (17.7)
Student	72 (18.0)	15 (16.7)	57 (18.4)
**Education level, n (%)**			
No formal	86 (21.5)	19 (21.1)	67 (21.6)
Primary	155 (38.8)	39 (43.3)	116 (37.4)
Secondary	98 (24.5)	20 (22.2)	78 (25.2)
Tertiary	61 (15.3)	12 (13.3)	49 (15.8)

**Types of surgeries:** the minor operation 246 (61.5%) and the major operation 154 (38.5%) with the most common being (Table 2): excision and biopsy 62 (15.5%), debridement 47 (11.8%), laparotomy 20 (5.0%), ORIF 18 (4.5%), OREF 17 (4.3), hemoroidectomy 17 (4.3%) and secondary closure 16 (4.1%) (Table 3).

**Table 2:** types of surgeries and cancellation of elective surgeries

Type of operation, n (%)	Total	Cancellation (%)	No cancellation (%)
	400 (100.0)	90 (22.5)	310 (77.5)
Minor	246 (61.5)	47 (52.2)	199 (64.2)
Major	154 (38.5)	43 (47.8)	111 (35.8)

**Table 3:** diversity of surgeries and cancellation of elective surgeries

Variable: surgery cancelled	Total	Yes (%)	No (%)
Surgery, n (%)	400 (100.0)	90 (22.5)	310 (77.5)
MAJOR	154 (38.5)	43 (47.8)	111 (35.8)
APR	1 (0.3)	0 (0.0)	1 (0.3)
Amputation	11 (2.8)	5 (5.6)	6 (1.9)
Bone drilling	1 (0.3)	1 (1.1)	0 (0.0)
Cholecystectomy	8 (2.0)	4 (4.4)	4 (1.3)
Sistrynk operation	1 (0.3)	1 (1.1)	0 (0.0)
Colostomy reversal	4 (1.0)	2 (2.2)	2 (0.6)
Grafting	10 (2.5)	3 (3.3)	7 (2.3)
Hemiarthroplasty	1 (0.3)	0 (0.0)	1 (0.3)
Ileostomy reversal	4 (1.0)	0 (0.0)	4 (1.3)
LAP	20 (4.3)	11 (5.6)	11 (5.8)
Mastectomy	3 (0.8)	2 (2.2)	0 (0.3)
Hernioplasty (mesh repair)	2 (0.6)	0 (0.0)	1 (0.6)
Myomectomy	2 (0.5)	1 (1.1)	1 (0.3)
OREF	17 (4.3)	2 (2.2)	15 (4.8)
ORIF	18 (4.5)	6 (6.7)	12 (3.9)
Polypectomy	1 (0.3)	0 (0.0)	1 (0.3)
Prostatectomy	6 (1.5)	2 (2.2)	4 (1.3)
Reduction+ fasciotomy	1 (0.3)	0 (0.0)	1 (0.3)
Urinary bladder repair	2 (0.5)	1 (1.1)	2 (0.6)
Rotational myocutaneous flap	1 (0.3)	0 (0.0)	1 (0.3)
TAH	10 (2.5)	3 (3.3)	7 (2.3)
Tendon repair	3 (0.8)	0 (0.0)	3 (0.9)
Thoracotomy	1 (0.3)	1 (1.1)	0 (0.0)
Thyroidectomy	7(1.8)	2 (2.2)	5(1.6)
Urethroplasty	7 (1.8)	3 (3.3)	4 (1.3)
Perineorrhaphy	2 (0.5)	1 (1.1)	1 (0.3)
Venus stripping	3 (0.8)	2 (2.2)	1 (0.3)
MINOR	246 (61.5)	47 (52.2)	199 (64.2)
Skin grafting	10 (2.5)	3 (3.3)	7 (2.3)
Hemorrhoidectomy	17 (4.3)	2 (2.2)	15 (4.8)
Tonsillectomy	8 (2.0)	0 (0.0)	8 (2.6)
SMC	16 (4.0)	5 (5.6)	11 (3.6)
SPC	2 (0.5)	0 (0.0)	2 (0.6)
STS	1 (0.3)	0 (0.0)	1 (0.3)
Saucerization	8 (2.0)	1 (1.1)	7 (2.3)
Scrotal exploration	1 (0.3)	0 (0.0)	1 (0.3)
Secondary closure	16 (4.1)	5 (5.5)	11 (3.4)
Sequestrectomy	1 (0.3)	0 (0.0)	1 (0.3)
Thiersch stich	1 (0.3)	0 (0.0)	1 (0.3)
Thoracosynthesis	1 (0.3)	1 (1.1)	0 (0.0)
Aural toilet (FB removal from ear)	1 (0.3)	0 (0.0)	1 (0.3)
BSO	9 (2.3)	1 (1.1)	8 (2.6)
Urethra bogging	2 (0.5)	0 (0.0)	2 (0.6)
Cauterization	1 (0.3)	0 (0.0)	1 (0.3)
Colporrhaphy	1 (0.3)	0 (0.0)	1 (0.3)
Bone Curettage	1 (0.3)	0 (0.0)	1 (0.3)
Debridement	47 (11.8)	9 (10.0)	38 (12.3)
Dorsal slit	1 (0.3)	0 (0.0)	1 (0.3)
EUA	6 (1.5)	1 (1.1)	5 (1.6)
Excision and biopsy	63 (15.8)	9 (10.0)	54 (17.4)
Feeding tube placement	1 (0.3)	1 (1.1)	0 (0.0)
Orchidopexy	2 (0.5)	1 (1.1)	1 (0.3)
POP and reduction	1 (0.3)	0 (0.0)	1 (0.3)
Partial sphincterotomy	2 (0.5)	0 (0.0)	2 (0.6)
Penile refashioning	1 (0.3)	0 (0.0)	1 (0.3)
Herniorrhaphy	23 (5.8)	2 (2.2)	20 (6.8)
Herniotomy	10 (2.5)	3 (3.3)	7 (2.3)
Hymenectomy	2 (0.5)	0 (0.0)	2 (0.6)
Mc Donald suture	1 (0.3)	0 (0.0)	(0.3)

Major surgery: APR: abdominoperineal resection; OREF: open reduction and eternal fixation; ORIF: open reduction and internal fixation; TAH: total abdominal hysterectomy: LAP: laparotomy. Minor surgery: aural toilet (foreign body removal from the ear); BSO: bilateral subscapular orchidectomy; EUA: evaluation under anesthesia; POP: plaster of Paris and reduction, SMC: safe male circumcision; SPC: supra pubic catheterization; STS: surgical toilet and suturing

**Factors associated with cancellation/deferring of elective surgery:** the current results did not show significant factors associated with the cancellation of elective surgeries at the bivariate analysis level; however, at multivariate level, major elective surgeries were cancelled 1.7 times more cancelled than minor surgeries [adjusted prevalence ratio 1.7 (95%CI: 1.07-2.73) and p-value: 0.024] (Table 4).

**Table 4:** bivariate and multivariate poisson regression analysis of cancellation of surgery with social demographic characteristics and other selected variables of the respondents

Variable	Cancellation		Bivariate		Multivariate	
	**Yes**	**No**	**Crude prevalence ratio (95%CI)**	**P-value**	**Adjusted prevalence ratio (95%CI)**	**P-value**
**Age group in years**						
20 and below	16 (15.5)	87 (84.5)	1.6 (0.91 - 2.97)	0.099	1.8 (0.91 - 3.71)	0.088
21 to 40	35 (25.5)	102 (74.5)	1.5 (0.78 - 2.8)	0.228	1.8 (0.84 - 3.97)	0.126
41 to 60	58 (24.5)	179 (75.5)	1.6 (0.91 - 2.74)	0.107	1.8 (0.93 - 3.62)	0.079
61 and above	16 (26.7)	44 (73.3)	1.7 (0.86 - 3.43)	0.126	2.1 (0.92 - 4.88)	0.078
**Sex**						
Male	52 (57.8)	189 (61.0)	1		-	-
Female	38 (42.2)	121 (39.0)	1.1 (0.73 - 1.68)	0.632	-	-
**Residence**						
Rural	51 (56.7)	196 (63.2)	1		-	-
Urban	39 (43.3)	114 (36.8)	1.2 (0.81 - 1.87)	0.322	-	-
**Profession**						
Private	9 (10.0)	36 (11.6)	1		-	-
Government	4 (4.4)	18 (5.8)	0.9 (0.28 - 2.95)	0.874	-	-
Self	45 (50.0)	144 (46.5)	1.2 (0.58 - 2.44)	0.633	-	-
Unemployed	17 (18.9)	55 (17.7)	1.2 (0.53 - 2.65)	0.687	-	-
Student	15 (16.7)	57 (18.4)	1 (0.46 - 2.38)	0.923	-	-
**Education level**						
No formal	19 (21.1)	67 (21.6)	1		-	-
Primary	39 (43.3)	116 (37.4)	1.1 (0.66 - 1.97)	0.642	-	-
Secondary	20 (22.2)	78 (25.2)	0.9 (0.49 - 1.73)	0.804	-	-
Tertiary	12 (13.3)	49 (15.8)	0.9 (0.43 - 1.83)	0.753	-	-
**Religion**						
Moslem	21 (23.3)	61 (19.7)	1		-	-
Christian	69 (76.7)	249 (80.3)	0.8 (0.52 - 1.38)	0.506	-	-
**Department**						
General	73 (81.1)	231 (74.5)	1		-	-
Orthopedic	10 (11.1)	39 (12.6)	0.8 (0.44 - 1.65)	0.63	-	-
Other (dental, ENT & GYN)	7 (7.8)	40 (12.9)	0.6 (0.29 - 1.35)	0.227	-	-
**Type of operation**						
Minor	47 (19.1)	199 (80.9)	1		1	
Major	43 (27.9)	111 (72.1)	1.5 (0.97 - 2.21)	0.072	1.7 (1.07 - 2.73)	0.024
**Anesthetic review**						
Done	30 (33.3)	141 (45.5)	1		1	
Not done	60 (66.7)	169 (54.5)	1.5 (0.96 - 2.31)	0.073	1.6 (0.98 - 2.46)	0.059

ENT: ear nose and throat; GYN: gynecology; CI: confidence interval

**Figure 2:**
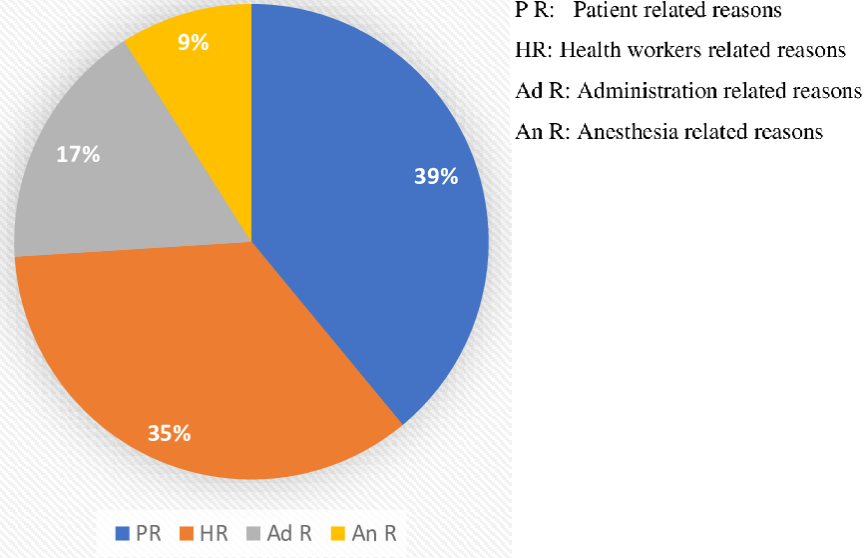
reasons of cancellation of elective surgeries in categories

**Causes of cancellation/deferring of elective surgery:** the most common causes of cancellation were patient related (39%), followed by surgeon related reasons (35%) and administrative issues (17%) (Figure 2). Among the patient related factors, the most common were the financial issues (23.3%), unavailability of the surgeon 15.5% and unfitness for surgery (14.4%) were the most common health worker related reasons for cancellation of elective surgery in this study (Table 5).

**Table 5:** distribution of reasons of cancelled surgeries at Kampala International University Teaching Hospital, 2019

Reason of cancellation	Category	Frequency	%
Health care workers related	Unavailability of the surgeon	14	15.5
	Patient not fit for surgery	15	16.7
	Patient needed further investigation	3	3.3
Patient related	Financial issues	21	23.3
	Patient declined the operation	11	12.2
	Patient didn't show up	3	3.3
Administration related	Lack of equipment	6	6.6
	No space for surgery	4	4.4
	Lack of blood	3	3.3
	Lack of oxygen	2	2.2
Anesthesia related	Patient not fit for anesthesia	5	5.5
	Failed cannulation of the patient	2	2.2
	Patient fed	1	1.1
Total		90	100

## Discussion

During the period under review (July 2019 - December 2019), 400 surgical interventions were planned, of which 90 (22.5%) were cancelled. Despite the fact that there is no acceptable range of cancellation, less than 5% is generally recommended [6], Although our findings are higher than the recommended range, they are within the range observed in other developing countries, which is between 10-45% [8,10-13]. The prevalence of cancellation in developed countries is reported to be between 0.37-28% [14,15].

In this study, the Department of General Surgery had the highest percentage of cancellation (81%), followed by orthopedics (10%) and gynecology 9% departments. This is different from the results published by Desta *et al*. in Ethiopia where cancellation of orthopedic cases was more prevalent (28.7%) followed by general surgery (17.8%) and gynecology (15.1%) [2]. In Tanzania, general surgery had the highest cancellation rate (31.5%) followed by orthopedics (25.5%) [12] which was different from what was found in Saudi Arabia [16] where orthopedics department was found to have the highest prevalence of cancellation at 33.9% and in Finland (31.8%) [17] of orthopedics cases among elective surgeries. However, Lonkoandea *et al*. found that the majority of cancelled surgeries were general surgery (80.4%), gynecology (11.7%) [13].

The age group 41-60, with a mean age of 36.7 (SD) years was the commonest among the participants in this study. This is different to studies done in Uganda and Sri Lanka in which the age groups of 10-19 years, 164 (41.0%) and 61-70 years (31.1%) were associated with more cancellations, respectively [8,18]. Pre-operative assessment a day before surgery might be the explanation behind this difference between studies.

In this study, the surgeries were grouped into minor operations 246 (61.5%) and major operations 154 (38.5%). These results are in line with those found by Hewawasam in Sri Lanka where the cancellations of major, intermediate and minor surgeries were 58.5%, 18.9%, 22.6% respectively [18]. In addition, major surgeries were more cancelled than minor surgeries, in this study. Proper assessment is needed prior to any surgery to avoid late cancellation which has an impact on socioeconomic aspects of surgical patients. However, much attention should be given to assessment of patients undergoing major surgery at least a day prior to operation.

The causes of cancellation among elective surgery were 39%, 35%, 17% and 9% for patient, Health workers, administration and anesthesia related reasons respectively. The results of this current study are similar to those found by Desta *et al*. in Ethiopia, which revealed that patient-related factors were a common reason for the cancellation of elective surgical cases accounting for 30% of cancellations [2] and Ezike *et al*. found in Nigeria found that the unavailability of the surgeon as the most common cause of cancelation at 35.8% [17]. However, our results of this study are different from the findings by Jonnalagadda *et al*. in a tertiary public teaching hospital in the Caribbean island of Barbados, who identified a number of administrative and infrastructural hindrances to the timely performance of elective surgical procedures [17]. This was also the case in another study carried out in Uganda by Ogwal *et al*. which revealed the cancellations mostly being inclined to health facility reasons (68.8%) and health care workers issues in (24.35%) [8].

In our study, the financial constraints contributed to the largest number of cancellations (23.3%) followed by unfitness of the patient to surgery (16.6%) while 15.5% was as a result of unavailability of the senior surgeon. The results are similar to those reported by Kaddoum *et al*. where the financial constraints were the most single cause of cancellation followed by incomplete medical work up [5]. Our findings are also similar to findings in Mulago Hospital in Uganda where the causes for the delay to surgery included lack of theatre space 44 (33.1%) cases, lack of blood to use in perioperative time 40 (30.1%) and patient related factors [19]. In a private hospital in Tanzania, it was found that the rate of cancellation of elective surgeries due financial issues was low (6.3%) [10] compared to findings in our study and this can be explained in part by the availability of the health assurance in Tanzania. Analysis of a government facility in central Uganda discovered that less than 5% of patients could access necessary surgical care without incurring catastrophic out-of-pocket expenditure [20]. Accessing surgical care in developing countries represent a substantial economic burden to a majority of the population [7,10].

Among the 90 cancelled cases only 46.6% benefited from pre-anesthetic assessment while 54.4% didn´t benefit from it. This rate is higher than that found in a study done in Burkina Faso by Lonkoandea *et al*. where pre-anesthetic assessment was not carried out for 7% of the patients who underwent the elective surgeries [13]. Earlier pre-anesthetic assessment of the surgical patient can allow more time to correct observed abnormal laboratory investigation results and to improve some uncontrolled medical conditions and therefore reduce cancellation rate [21,22]. Pre-admission anesthetic consultations at these clinics could not only improve efficiency of theatre use but also reduce the duration of hospital stay and hospital costs [21,22].

During data collection of this study, a follow up of all prospective theatre lists was done by assigning a research assistant to review the lists before submission to the theatre team to avoid bias of selection and result; however, this study was not evaluating the financial income of the patient during data collection which could have informed the relationship between cancellation of elective surgeries and level of financial income.

## Conclusion

The cancellation of elective surgeries is still high in our setting. Most cancellations were avoidable and therefore there is a need for more audits to mitigate this issue. In order to enhance cost effectiveness and efficiency, efforts should be made to prevent unnecessary postponement through careful planning aimed at proper scheduling, pre-anesthetic evaluation and perioperative preparation, provide adequate information for the scheduled patient, providing necessary operating room equipment by hospital administrators and other stakeholders and clear communication with operating room team. We suggest for future research on cancellation of elective surgeries should compare private and public health facilities and analyze the role of health financing mechanisms.

### What is known about this topic

- The cancellation of elective operations on the intended day and its effect in the urban health setting;
- Most studies on cancellation are done in public hospitals where the government bear the cost of the operations.

### What this study adds

- This study is adding data about the prevalence and causes of cancellations in the rural private health setting;
- This study is highlighting the deer need of introducing of health assurance for all Ugandans;
- Quality improvement strategies to be designed to improve on communication between health workers, patients and administration and also pre-anesthetic assessment to be done as a must.
